# Knowledge Sharing Among Healthcare Practitioners: Identifying the Psychological and Motivational Facilitating Factors

**DOI:** 10.3389/fpsyg.2021.736277

**Published:** 2021-12-14

**Authors:** Su-Ying Wu, Wei-Tsong Wang, Ming-Hsuan Hsiao

**Affiliations:** ^1^Institute of Information Management, National Cheng Kung University, Tainan City, Taiwan; ^2^Kaohsiung Medical University Hospital, Kaohsiung Medical University, Kaohsiung, Taiwan; ^3^Department of Industrial and Information Management, National Cheng Kung University, Tainan City, Taiwan

**Keywords:** knowledge sharing, psychological empowerment theory, psychological ownership theory, self-determination theory, healthcare organization

## Abstract

There exists a lack of an understanding of how to facilitate knowledge sharing (KS) behaviors in healthcare organizations. This study is among the first to specifically address this issue through synthesizing psychological ownership (PO), self-determination theory, and psychological empowerment (PE) theory. This study developed a research model that described the impact of the psychological and motivational facilitating factors, including autonomous motivation, user PE, and PO on knowledge sharing intention (KSI) and knowledge sharing behavior (KSB). Data collected from 343 healthcare professionals were analyzed using the technique of partial least squares (PLS) to validate the research model. The results indicated that user PE, organization-based PO, and autonomous motivation all had significant direct/indirect positive effects on KSI and KSB as we hypothesized. Surprisingly, knowledge-based PO had a significant positive effect on KSI, which contradicted our original hypothesis. The implications for theory and for practice, limitations, and future research directions are discussed accordingly.

## Introduction

Knowledge has long been recognized as the primary source of organizational growth and sustainable competitive advantage (Alsharo et al., 2017; Stenius et al., 2017; Li and Kang, 2019; Mc Evoy et al., 2019). Organizations often face challenges and competition in uncertain environments in order to stand out in a highly competitive industry, relying heavily on how effectively critical knowledge is being shared among employes (Curtis and Taylor, 2018; Al-Kurdi et al., 2020). Prior studies have indicated that efficient knowledge sharing (KS) plays a key role in the success of knowledge management (KM) initiatives (Al-Kurdi et al., 2020). It may offer organizations the leverage they need for developing competitive advantages (Wu and Lee, 2017; Hameed et al., 2019; Hao et al., 2019). Therefore, determining how to effectively facilitate KS practice in organizations is of great value. Healthcare organizations are highly knowledge-intensive organizations that involve a wide range of professionals with expertise in different disciplines who are required to pay attention to constant updates in relevant technologies and areas of knowledge. In the dynamic healthcare services process, employes, including physicians, nurses, pharmacists, medical technologists, rehabilitation therapists, social workers, dieticians, radiologic technologists, information engineers, and administrative managers, often have to leverage their expertise to collaborate with professionals from various fields and to solve problems cooperatively. Therefore, healthcare practitioners have a strong demand for KS.

However, even though the factors affecting KS have been extensively investigated, gaps remain in the existing research on knowledge sharing behavior (KSB). First, a significant amount of research has explored the factors affecting KSB based on the social exchange theory, which proposes that human behavior is driven by individual expectations of the costs and benefits inherent in interpersonal exchanges (Yan et al., 2016; Wu and Lee, 2017; Curtis and Taylor, 2018; Qian et al., 2019). However, focusing on understanding individuals’ willingness to share knowledge for the purpose of the exchange of benefits may not be sufficient to explain proactive KSB (Kang et al., 2017). Psychological empowerment (PE) can increase an individual’s inherent level of task motivation and can lead to that person’s ability to actively and consistently accomplish organizational goals due to individual perceptions or evaluations of the meaning of work (Thomas and Velthouse, 1990; Spreitzer, 1995), which is considered to be a positive motivational orientation (Kang et al., 2017). Gaining a better understanding of proactive KS requires taking into account an active motivational orientation (Kang et al., 2017), such as PE. Furthermore, it is critical to implement information systems (ISs) to process data in order to provide information and knowledge that support high-quality, efficient patient care, as well as administrative managerial tasks in healthcare environments (Haux, 2006; Ljubicic et al., 2020). Such complexes or systems can be briefly called health information systems (HISs), including electronic health records (EHRs), hospital operations management, supportive healthcare policy decision systems (Goldschmidt, 2005; Khubone et al., 2020), and modules related to KM, such as online learning platforms, document-management modules, healthcare data warehouses, electronic knowledge repositories, desktop virtualization, and cloud tools used for storing and sharing. In the context of these collaborative communication channels (i.e., HISs), user PE provides a rationale for understanding the process and consequences of employes’ efforts to exert dominance and influence the outcomes of their tasks (Wang et al., 2019). From the standpoint of person-environment interaction, PE is a context-oriented concept, where contextual influences should not be ignored (Zimmerman, 1990). We incorporate the context of HIS usage into user PE, exploring whether user PE affects KS and facilitates the effective integration and sharing of the knowledge and experience of employes. This is a crucial issue for healthcare organizations and worth the effort required to conduct a comprehensive investigation.

Second, many studies have investigated individual motivation that leads to KSB, including intrinsic motivation (e.g., self-efficacy, altruism, or enjoyment from helping), extrinsic motivation (e.g., financial rewards, reciprocity, or reputation). Nevertheless, it may be difficult to understand the overall quality of motivations in terms of the level of autonomy involved. This study is an attempt to close this gap in the literature by focusing on the controlled-to-autonomous continuum of motivations proposed in SDT (self-determination theory) (Gagne and Deci, 2005), rather than treating one’s behavioral motives as an intrinsic/extrinsic binary. We directly incorporated the construct of autonomous motivation into our research framework and measured it using the relative autonomy index (RAI) based on types of motivation proposed in SDT (Cockrell and Stone, 2010). RAI is a summarized holistic score of the quality of motivation (autonomy) that can appropriately represent the degree to which an employe autonomously engages in KS. This makes it possible to better understand how different degrees of autonomy affect employe KSB.

Third, in addition to attempting to explore the proactive motivation toward KS, it is important to understand the barriers to KS. Based on the nature of self-interest, employes may be afraid of losing their knowledge power and their unique value within an organization (Bock et al., 2005; Alsharo et al., 2017); they may also be reluctant to share the target of ownership (e.g., knowledge) with one another or may feel they need to retain exclusive control over it (Pierce et al., 2001, 2003; Ford and Staples, 2010), which may have a negative impact on KSB. This state could be explained by the theory of psychological ownership (PO). PO is a mental state in which an individual feels possessiveness tied to objects (Pierce et al., 2001, 2003; Dawkins et al., 2017). The effects of PO on KSB seem complicated since some are positive, like organization-based psychological ownership (OPO), and others are negative, such as knowledge-based psychological ownership (KPO) (Peng and Pierce, 2015). Although PO has been considered an important organizational phenomenon (Pierce et al., 2001; Dawkins et al., 2017), the literature link to behavior (e.g., KSB) is rather fragmented and scarce. Previous studies have paid little attention to the psychological contradictions inherent in KS, and there is also a lack of understanding of the impact of the interaction between PO and positive motivation on KS. To fill this gap, we adopt the two types of PO (OPO and KPO) to better understand how organizations influence employe perceptions of PO, in turn resulting in effective KS.

In summary, to bridge these gaps, this study adopted SDT as the main underlying theory and integrated PE and PO theories to develop a research model that present the relationships among KS motivations, knowledge sharing intention (KSI), and KSB. Focusing on the potential psychological and motivational factors, including user PE (intrinsic task motivation), autonomous motivation (the degree of autonomy on the motivational continuum), PO (being psychologically tied to an object as an extension of one’s identity, such as the organization or knowledge), KSI (a willingness to exert effort to achieve goals) to explore the effects of these factors on KSB among healthcare practitioners, as well as the moderating role of autonomous motivation in order to advance our understanding of KS and provide useful insights into initiating appropriate measures to achieve effective KS practices.

## Theoretical Background and Hypotheses Development

Knowledge will become more valuable through sharing. KS not only allows personal knowledge to accumulate, but also enhances the competitiveness of enterprises. KS plays a crucial role in the knowledge-based economy era (Wu and Lee, 2017). It is an employe behavior that is a key dynamic for the success of knowledge-intensive organizations (Ding et al., 2017; Stenius et al., 2017; Mc Evoy et al., 2019; Al-Kurdi et al., 2020), such as healthcare organizations. Although the issue of KS has been widely discussed in the growing literature on the topic (Han et al., 2019; Qian et al., 2019; Al-Kurdi et al., 2020; Jiang and Xu, 2020), the number of studies contributing to the interpretation of and solutions to developing KSB in healthcare organizations is still limited. Accordingly, determining how to encourage employes to willingly share knowledge and in turn improve performance and the quality of medical services is a key issue for healthcare organizations.

### Knowledge Sharing Intention and Knowledge Sharing Behavior

Existing literature has indicated that KS is a process of multi-directional exchanges of individual knowledge (Hsu et al., 2007; Gagne, 2009; Li and Kang, 2019). It is an enabler for the development of new knowledge and helps in collaborating with others to solve problems (Matic et al., 2017; Li and Kang, 2019). We define KSB as a set of behaviors in which work-related knowledge, suggestions, ideas, experiences, expertise, and skills are shared and exchanged in healthcare organizations to help leverage the skills, knowledge, and best practices of professional staff (Chang et al., 2015; Zhang et al., 2017; Curtis and Taylor, 2018; Hao et al., 2019; Wang et al., 2019; Kakhki et al., 2020).

According to the theory of reasoned action (TRA) and theory of planned behavior (TPB), behavioral intention (i.e., KSI) is considered to be the most important determinant of behavior (Ajzen and Fishbein, 1977; Ajzen, 1991) and an immediate predictor of KSB (Bock et al., 2005; Curtis and Taylor, 2018). If individuals have a stronger intention to engage in a behavior, they are more likely to act accordingly (Ding et al., 2017; Hoseini et al., 2019; Obrenovic et al., 2020). In this study, we define KSI as the desire and willingness of healthcare professionals to share their knowledge, ideas, suggestions, experiences, and skills with others in healthcare organizations.

Many prior studies have provided empirical evidence supporting a positive correlation between KSI and KSB (Safa and Von Solms, 2016; Matic et al., 2017; Stenius et al., 2017; Hoseini et al., 2019; Al-Kurdi et al., 2020; Kakhki et al., 2020), as shown in Table 1. However, the findings of some studies were inconsistent with those of the majority of the other studies on the topic (Chatzoglou and Vraimaki, 2009; Dey and Mukhopadhyay, 2018). Also, some studies only adopted the KSI construct to represent KSB (Zhang et al., 2017; Wang and Chang, 2018; Han et al., 2019), which may not help determine the relationship between intention and real behavior. Investigating how KSI affects KSB would be helpful in terms of understanding KS in the highly uncertain work context of healthcare organizations. Therefore, we developed the following hypothesis:

*H1: KSI positively influences KSB.*

**TABLE 1:** A summary of empirical research on KSB.

No	Author	Sample size	Sample characteristics	Result KSI- > KSB
1	Al-Kurdi et al. (2020)	257	Higher Education Institutions, United Kingdom	Positive significant (***)
2	Kakhki et al. (2020)	208	Librarians of public libraries, Iran	Positive significant (**)
3	Hoseini et al. (2019)	161	Users of MSN, Iran	Positive significant (***)
4	Matic et al. (2017)	873	50 service and manufacturing organizations in the public and private sectors, Serbia	Positive significant (***)
5	Stenius et al. (2017)	200	A large public-sector organization, Finland	Positive significant (***)
6	Yeon et al. (2016)	286	Members of the Biology Research Information Center, South Korea	Positive significant (***)
7	Safa and Von Solms (2016)	482	Banking, insurance, e-Commerce and education organizations, Malaysia	Positive significant (**)
8	Han et al. (2019)	396	IT companies, South Korea	None
9	Wang and Chang (2018)	286	High tech, service, financial, manufacturing industries, Taiwan	None
10	Zhang et al. (2017)	443	Three famous online health communities, China	None
11	Chatzoglou and Vraimaki (2009)	276	State-owned and private bank branches, Greece	Positive, No significant
12	Dey and Mukhopadhyay (2018)	246	Managers of private sector firms, Indian	Positive, No significant

**p < 0.01; ***p < 0.001.

### Psychological Ownership

Psychological ownership is based on the psychological theory of possession, which involves feelings of psychological possession of objects (Pierce et al., 2001, 2003; Dawkins et al., 2017). The sense of possession may be decoupled from legal ownership of targets. Even without real property rights, employes can develop PO via mental experiences (Hameed et al., 2019). Such feelings of ownership can be developed toward material (e.g., the organization, products) and immaterial (e.g., knowledge) objects (Pierce et al., 2003; Pittino et al., 2018).

Psychological ownership is a key emerging construct in the area of organizational behavior (Dawkins et al., 2017), but little is known of the underlying mechanism leading to KSB. While employes may have a deep emotional investment in their profession in healthcare organizations, this sense of possession toward targets is either an obstacle to or an incentive for KS. An understanding of the relationship of PO with organizations and knowledge would be helpful to clarify the positive and negative effects on KSB among healthcare practitioners and to enrich PO findings in the KS literature. Therefore, this study conceptualizes PO from two perspectives in the framework: OPO and KPO. OPO is the psychological state in which employes develop possessive feelings toward healthcare organizations, whereas KPO is the psychological state in which employes develop feelings of possession toward knowledge in healthcare organizations. Most PO studies have solely focused on OPO (Dawkins et al., 2017). However, Wang and Noe (2010) indicate that individual perceptions of KPO are rarely studied and need to be better understood.

Research findings have suggested that the cognition of OPO is an essential part of the employe-organization relationship (Hameed et al., 2019), reflecting the sense of responsibility and accountability for ownership objectives toward the organization (Avey et al., 2009; Dawkins et al., 2017; Pittino et al., 2018). It can arouse an altruistic spirit and in turn stimulate positive work-related outcomes, such as KSI and KSB (Pierce et al., 2001; Han et al., 2010). Existing empirical studies have found that OPO is positively related to KSB (Li et al., 2015; Hameed et al., 2019), and it has been deemed one of the critical antecedents of KSB (Han et al., 2010). Based on the above discussion, we propose the following hypothesis:

*H2a: OPO positively influences employe KSI.*

Relative to OPO, few studies have examined the antecedents and consequences of employe perceptions toward KPO. Excessive feelings of possession may trigger negative sides of possessiveness, in turn inhibiting the implementation of KSB (Pierce et al., 2003). Employes may wish to have exclusive control over the ownership of knowledge or may be afraid to lose their unique value within the organization (Bock et al., 2005) and are thus unwilling to share knowledge with others. Perceptions of ownership may motivate an individual to protect or defend knowledge as an ownership right (Pierce et al., 2001), leading to resistance to sharing knowledge. Related empirical studies have obtained mixed results in attempts to determine the relationship between KSB and KPO. For example, a study by Ford and Staples (2010) showed that KPO is positively associated with the willingness to share; however, a negative impact of KPO on KS was revealed by Li et al. (2015). We developed the following hypothesis concerning the negative impacts of KPO (Pierce et al., 2003).

*H2b: KPO negatively influences employe KSI.*

### Psychological Empowerment

The empowerment theory provides a comprehensive theoretical foundation that pictures how organizations build a helpful environment (e.g., HIS-assisted foundation for work-related goals) (Spreitzer, 1995; Wang et al., 2019). Empowerment is a multifaceted concept (Thomas and Velthouse, 1990; Spreitzer, 1995) from a psychological perspective. It focuses on personal perceptions and empowering experiences and suggests that intrinsic task motivation can be boosted (Thomas and Velthouse, 1990; Spreitzer, 1995). Since employes are the key players implementing KS activities in an organization (Wu and Lee, 2017), it is important to understand their psychological motivations related to KS. PE theory can accurately account for an individual’s active motivational orientation. Kang et al. (2017) applied user empowerment with the use of knowledge management systems to explore employe behavior related to knowledge contribution and knowledge seeking. IS can support the KS process and is an important facilitator that motivates employes to participate in the process (De Almeida et al., 2016). However, in healthcare organizations, the use of HISs is inevitable for employes who need to use such systems to improve task performance and collaborate with others. In the context of HIS usage, exploring the development of user PE and its impact on KS makes it possible to better understand the application of this active motivational orientation in healthcare organizations. Thus, this study conceptualizes the extension of PE, defining user PE as an employe’s active motivational orientation toward using HISs at work as well as roles manifested in four dimensions: the meaning of HIS usage, user competence, user self-determination, and the impact of HIS usage on healthcare organizations (Spreitzer, 1995; Kang et al., 2017). The meaning of HIS usage is defined as the value of an employe related to a work goal or purpose in the context of HIS use. User competence refers to a belief in an employe’s ability to use HIS to perform tasks with skill. User self-determination indicates the degree to which an employe can freely control the work in terms of HIS usage. Finally, the impact of HIS usage is defined as the extent to which an employe can affect outcomes at work based on HIS use.

Based on PO, empowered employes have a high sense of self-efficacy and self-identify, in turn giving them the ability to have a significant impact on tasks and circumstances with the use of HISs and to be proactive in terms of fulfiling their responsibilities (Spreitzer, 1995; Benton and Magnier-Watanabe, 2014), which in turn enhances cohesion and their sense of belonging to an organization. These traits of empowered employes may lead to PO (efficacy, self-identity, and belongingness) as proposed by PO theory (Pierce et al., 2001, 2003; Van Dyne and Pierce, 2004). Also, feelings of PO create a powerful sense of responsibility and accountability for ownership objectives (Avey et al., 2009; Dawkins et al., 2017; Pittino et al., 2018). Thus, it is expected that employes who sense higher levels of user PE may perceive higher levels of PO (OPO and KPO). Bendermacher et al. (2019) empirically demonstrated that PE and PO are positively and significantly correlated. Furthermore, Peng and Pierce (2015) suggested that PE is a factor that enhances employes’ feelings of ownership and satisfaction, that motivates them and stabilizes their mental state. Based on the above discussion, the following hypotheses were developed:

*H3a: User PE positively influences OPO.*

*H3b: User PE positively influences KPO.*

Many studies have validated the positive impact of PE on employe behavior (e.g., job satisfaction, job engagement, productivity, and task performance) in the workplace (Spreitzer, 1995; Dust et al., 2014). KS is a proactive behavior (Kang et al., 2017) that cannot be coerced. It can, however, be encouraged and facilitated (Bock et al., 2005). On the other hand, PE is an active motivational orientation (Kang et al., 2017), which leads to a better understanding of the factors that motivate proactive behavior like KSB. HISs provide critical support for managing healthcare activities related to the collection, organization, storage, and communication of information and knowledge, in turn improving quality of care and coordination among employes (Fichman et al., 2011). HISs facilitate communication among various healthcare professionals through the shared knowledge repositories. In the context of HIS usage, user PE serves as a specific, active form of work motivation for employes, which can enhance their self-efficacy, increase their confidence in their abilities, and lead them to believe that their knowledge can help resolve job-related problems and improve work efficiency (Kang et al., 2017). This may lead employes to be willing to strive for continuous improvement of functioning HISs through high levels of involvement and increase the likelihood of their sharing knowledge in order to support their tasks, intrinsically motivating them to actively accomplish organizational goals (Thomas and Velthouse, 1990; Spreitzer, 1995). It is important to advance an understanding of factors motivating and driving KS surrounding the use of HISs in healthcare settings. Therefore, the following hypothesis is developed:

*H3c: User PE positively influences employe KSI.*

### Autonomous Motivation

At present, SDT (Deci et al., 1989) is a popular KS motivation theory. SDT has received widespread attention to explain behavioral motivations in the education, healthcare, and sports domains (Gagne and Deci, 2005).

Self-determination theory spotlights how different types of motivation reflect levels of autonomy, where motivation can be divided into amotivation (lack of motivation) or a controlled-to-autonomous continuum, which ranges from extrinsic motivation (controlled) to intrinsic motivation (autonomous) (Gagne and Deci, 2005). Extrinsic motivation can be categorized into four different levels of progressively increasing autonomy: external regulation, introjected regulation, identified regulation, and integrated regulation, which differ in terms of their underlying levels of autonomy (Gagne and Deci, 2005). External regulation (controlled motivation) refers to behaviors controlled by specific external contingencies, such as obtaining rewards or avoiding punishment (Gagne and Deci, 2005). Introjected regulation (moderately controlled motivation) refers to an individual behaving according to the beliefs and expectations of the self or others in order to decrease negative feelings (e.g., guilt and shame) or to buttress positive feelings, such as pride (Deci and Ryan, 2000). Identified regulation (moderately autonomous motivation) refers to where individuals are motivated by a conscious perception of the value of a behavior and willingly accept it as their own (Gagne and Deci, 2005; Wang, 2016). Integrated regulation (autonomous motivation) refers to individuals being motivated by recognizing that the value of a behavior is consistent their own values and goals (Deci and Ryan, 2000; Wang, 2016). However, intrinsic motivation reflects the purest expression of autonomy, which is driven by perceiving that the behavior itself is interesting, pleasant, and inherently satisfying (Deci and Ryan, 2000; Wang, 2016).

Previous research indicates that more attention should be paid to the degree of influence of the controlled-to-autonomous continuum (Gagne and Deci, 2005; Wang, 2016). To reduce the number of variables being evaluated and to increase the parsimony of the model, we measured autonomous motivation referring to the RAI (i.e., we integrated four motivational constructs into a single index of autonomous motivation) proposed by Grolnick and Ryan (1987) to better understand the overall effect of employe motivation on KSB. The RAI was calculated using the following equation (Grolnick and Ryan, 1987; Cockrell and Stone, 2010):


$$\begin{array}{c} \mathrm{RAI}=\left(2*\mathrm{intrinsic}\mathrm{motivation}+\mathrm{identified}\mathrm{regulation}\right)-\\ \left(\mathrm{introjected}\mathrm{regulation}+2*\mathrm{external}\mathrm{motivation}\right). \end{array}$$

A higher RAI score indicated that the individual’s behavior is autonomously motivated to a greater degree, whereas a lower the score indicated that the individual’s behavior is more controlled.

Psychological empowerment can be considered a motivational cognitive state, representing employes’ motivational orientation along with the authoritative power required to perform work (Thomas and Velthouse, 1990; Kang et al., 2017). HISs support employes’ ability to efficiently perform tasks with the skills necessary to improve the quality and efficiency of health services, along with increased competence related to performance. HISs also makes it possible for employes to collaborate more efficiently with others across diverse practices (Fichman et al., 2011; Ljubicic et al., 2020). Employes may recognize the value and benefits of using an HIS, which in turn inspires them to proactively exploit such systems to their full potential in order to complete tasks. In the context of HIS usage, empowered employes are granted the discretion to schedule their work and have more autonomy to determine how they want to do things. This raises their levels of self-efficacy and promotes task initiation and persistence (Conger and Kanungo, 1988; Kang et al., 2017). It can also drive highly autonomous intrinsic task motivation and exert a significant impact on increasing satisfaction with the three basic human psychological needs (autonomy, competence, and relatedness) proposed in the SDT, which boosts individual enjoyment of activities and supports the autonomous self-regulation of behaviors that facilitate KSB (Deci and Ryan, 2000). Thus, user PE is expected to be positively related to autonomous motivation. Accordingly, we propose the following hypothesis:

*H3d: User PE positively influences autonomous motivation.*

Motivation stems from personal expectations of beneficial outcomes, which encourages willingness to engage in certain behaviors (Zhang et al., 2017), such as KS. It is unlikely to forecast people’s behavior and performance without sufficient motivation (Hwang et al., 2018). However, SDT does help scholars gain an understanding of the motivational basis of individual behavior. Many studies exploring KS are based on SDT, and their findings indicate that autonomous motivation plays an important role in facilitating KSB (Gagne, 2009; Wang, 2016; Stenius et al., 2017). Wang (2016) found that when employes have more autonomy, they are more willing to share knowledge. Gagne (2009) presented a model of KS motivation and proposed that autonomous motivation is positively related to KSI. Stenius et al. (2017) found that autonomous motivation was one of the key predictors of KS. Consequently, we propose the following hypotheses:

*H4a: Autonomous motivation positively influences employe KSI.*

*H4b: Autonomous motivation positively influences employe KSB.*

This study investigates the potential antecedents of KSB, including user PE, PO, autonomous motivation, and KSI. However, based on a review of previous research, the existing research has not shed much light on the interactions among these predictors. This research is intended to fill the gap by exploring autonomous motivation as a strategic instrument playing a moderating role. When serving as a moderator, autonomous motivation moderates the relationship between PO and KSI depending on the level of autonomous motivation, as well as whether employes react differently when experiencing OPO or KPO, and in turn determines their level of KSI. Employes with higher levels of autonomous motivation may have a greater sense of freedom and discretion at work, and can thus control the manner in which they complete tasks. They have confidence related to controlling their surrounding environment and thus develop a sense of responsibility and accountability. In this situation, we believe that autonomous motivation has the potential to strengthen the relationship between PO (including OPO and KPO) and KSI, because it cultivates the awareness of PO by satisfying the need for efficacy, self-identity, and belongingness (Pierce et al., 2001, 2003). The higher the level of autonomous motivation is, the stronger the sense of OPO, and the sturdier the relationship is between employes and organizations. Thus, more KSI will be encouraged to facilitate KSB. Thus, we argue that the effect of OPO on KSI is likely to be stronger in the context of higher levels of autonomous motivation than it will be in contexts with lower levels of autonomous motivation. On the other hand, when employes have high levels of autonomous motivation, this suggests that they have more autonomy, which may lead them to have greater control over knowledge, perhaps in order to defend knowledge ownership rights or preserve their unique value in the organization, thereby reducing their intention to share knowledge (Li et al., 2015). Therefore, we suggest autonomous motivation may strengthen the negative relationship between KPO and KSI. Accordingly, we propose the following hypotheses:

*H4c: Autonomous motivation positively moderates the positive relationship between OPO and KSI.*

*H4d: Autonomous motivation positively moderates the negative relationship between KPO and KSI.*

**FIGURE 1:**
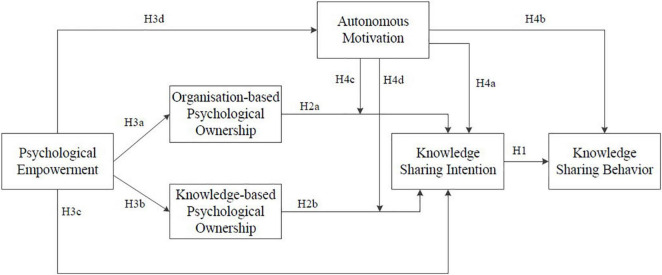
The research model.

Based on the discussion above, the proposed research model is shown in Figure 1.

## Research Method

### Development of the Instruments

To conduct an effective survey, 48 items related to the six constructs of the research model were adapted and refined from the existing literature according to the specific focus of this study to ensure content validity (corresponding sources for each structure can be found in the Supplementary Appendix). All items were measured using seven-point Likert scales, ranging from (1) strongly disagree to (7) strongly agree. Furthermore, the demographic variables included gender, age, education, and tenure as the control variables in this study to take into account the potential effects of these characteristics on the respondents’ tendency to share knowledge.

### Data Collection

Prior to the formal data collection process, all measurement items were pilot-tested with 39 randomly selected employes from different hospitals with experience of HIS usage. The internal consistency and reliability were examined using a Cronbach’s alpha coefficient analysis. The results (ranging from 0.72 to 0.95) showed that all first-order constructs reached the recommended level of 0.7 (Hair et al., 2017). Thus, no changes were made to the questionnaire.

Data for the study were collected from the employes of three large teaching hospitals that were under the supervision of a prestigious research-centered medical university in Taiwan, and all these three hospitals have implemented HIS to support the provision of various healthcare services. We focused on investigating KSB of the employes with experience with HIS usage, and we thus randomly distributed 450 questionnaires with the consent of the supervisor of the employes in these three hospitals. Participation in the survey was voluntary, and the confidentiality of the identities of the survey respondents was ensured. The survey respondents were requested to answer questions based on their experience with using KM-related modules of the HIS (e.g., online learning platforms, document management modules, healthcare data warehouses, electronic knowledge repositories, virtual desktop infrastructure, and cloud tools for storing and sharing). At the end of the data collection process, 430 responses were received. Eventually, 87 responses were excluded because some respondents failed to answer all the questions in the survey, had a systematic answering pattern, or failed to pass the check of the reverse questions that were deliberately included in the questionnaire. Finally, a total of 343 valid questionnaires were obtained, achieving a valid return rate of 79.8%. Table 2 provides the demographic information for the respondents. Of the respondents, 74.3% participants were female, and 25.7% were male. The gender distribution in the study was similar to that reported by the Executive Yuan, Taiwan on the gender statistics of medical staff (Executive-Yuan, 2020). The majority of the respondents were 31–40 years of age (40.5%), served in the organization for 16 years and above (26.5%), and had a bachelor’s degree (69.7%).

**TABLE 2:** Respondent demographics.

Characteristics		Number	Percentage (%)
Gender	Male	88	25.7
	Female	255	74.3
Age	21–30	83	24.2
	31–40	139	40.5
	41–50	101	29.4
	51–60	20	5.8
Education	College	12	3.5
	University	239	69.7
	Postgraduate	92	26.8
Tenure	<1	29	8.5
	1–3	45	13.1
	4–6	51	14.9
	7–9	36	10.5
	10–12	38	11.1
	13–15	53	15.5
	> = 16	91	26.5

To assess for potential non-response bias, an independent samples *t-*test was performed on the demographic variables (including gender, age, education, and tenure) by comparing early and late responders. The results showed that these two data sets had no statistically significant differences with respect to gender (*p* = 0.31), age (*p* = 0.37), education (*p* = 0.81), and tenure (*p* = 0.47), suggesting that the non-response bias was unlikely to be a serious problem in this study.

### Data Analysis Method

A partial least squares (PLS) analysis, which is a variance-based structural equation modeling (SEM) technique, was used to validate our research model. Based on the flexibility of the PLS terms of model measurement, it can handle reflective and formative types of latent variables (Hair et al., 2017) and also can be used to model the multi-dimensional items of latent constructs. In our research model, user PE was modeled as a second-order formative construct. Furthermore, PLS does not have strict requirements for data distribution and works well for small-to-medium sample sizes (Hair et al., 2017). Accordingly, we determined PLS to be a suitable technique for our data analysis procedures, and SmartPLS 3.3 software was used to analyze the research model.

## Data Analysis and Results

### Measurement Validation

Convergent and discriminant validity were first evaluated to validate the research model. Convergent validity can be assessed by inspecting the factor loadings of the indicators, composite reliability (CR), and average variance extracted (AVE) from the measures (Hair et al., 2017). Three items, EXM1, OPO7, and KPO6 were discarded due to their having a low factor loading. After deleting these items, all remaining factor loadings were statistically significant, and all estimates (ranging from 0.65 to 0.95) were larger than the suggested value of 0.6 (Chin, 1998). The Cronbach’s alpha values (ranging from 0.71 to 0.95) of all constructs were above the suggested threshold of 0.7. The CR statistics of all constructs ranged from 0.83 to 0.96, which were all greater than the recommended level of 0.7 (Hair et al., 2017). Meanwhile, the AVE statistics of all of the constructs ranged from 0.6 to 0.87, which were also above the cutoff value of 0.5 (Fornell and Larcker, 1981). These results are shown in Table 3, where they indicate acceptable construct reliability.

**TABLE 3:** Reliability and convergent validity of the measurement model.

Construct	Indicator	Factor Loading *	Cronbach’s alpha coefficient	Composite Reliability (CR)	Average Variance Extracted (AVE)
Meaning (PEM)	PEM1	0.92	0.92	0.95	0.87
	PEM2	0.94			
	PEM3	0.94			
Competence (PEC)	PEC1	0.94	0.92	0.95	0.86
	PEC2	0.94			
	PEC3	0.90			
Self-determination (PESD)	PESD1	0.94	0.93	0.95	0.87
	PESD2	0.94			
	PESD3	0.92			
Impact (PEI)	PEI1	0.91	0.92	0.95	0.86
	PEI2	0.93			
	PEI3	0.95			
Organization-based psychological ownership (OPO)	OPO1	0.70	0.88	0.90	0.61
	OPO2	0.82			
	OPO3	0.72			
	OPO4	0.74			
	OPO5	0.84			
	OPO6	0.84			
Knowledge-based psychological ownership (KPO)	KPO1	0.73	0.83	0.88	0.60
	KPO2	0.66			
	KPO3	0.78			
	KPO4	0.85			
	KPO5	0.82			
Knowledge Sharing Behavior (KSB)	KSB1	0.91	0.94	0.95	0.80
	KSB2	0.87			
	KSB3	0.92			
	KSB4	0.89			
	KSB5	0.88			
Knowledge Sharing Intention (KSI)	KSI1	0.84	0.95	0.96	0.82
	KSI2	0.92			
	KSI3	0.94			
	KSI4	0.93			
	KSI5	0.89			
External Motivation (EXM)	EXM 2	0.87	0.71	0.83	0.62
	EXM 3	0.81			
	EXM 4	0.68			
Introjected Regulation (IJR)	IJR 1	0.65	0.79	0.86	0.61
	IJR 2	0.84			
	IJR 3	0.81			
	IJR 4	0.80			
Identified Regulation (IDR)	IDR 1	0.72	0.87	0.91	0.72
	IDR 2	0.91			
	IDR 3	0.88			
	IDR 4	0.87			
Intrinsic Motivation (INM)	INM 1	0.86	0.92	0.94	0.80
	INM 2	0.90			
	INM 3	0.91			
	INM 4	0.90			

*All factor loadings of the individual items were statistically significant (p < 0.001).

Finally, discriminant validity (Fornell-Larcker criterion) was assessed by examining the square roots of the AVE statistics (Fornell and Larcker, 1981). The results shown in Table 4 indicate that the square roots of the AVEs for each first-order reflective construct were greater than the correlations between the constructs. We also employed the heterotrait-monotrait (HTMT) ratio of correlations for evaluating the discriminant validity (Henseler et al., 2015). The results shown in Table 5 indicate that all HTMT values were below the cutoff value of 0.85 (Henseler et al., 2015). These results support the discriminant validity of the measurement model.

**TABLE 4:** Discriminant validity – Fornell-Larcker criterion for the measurement model.

	PEM	PEC	PESD	PEI	KSB	KSI	KPO	OPO	EXM	IJR	IDR	INM
PEM	0.93											
PEC	0.53	0.93										
PESD	0.45	0.62	0.93									
PEI	0.23	0.32	0.49	0.93								
KSB	0.48	0.46	0.52	0.45	0.89							
KSI	0.52	0.56	0.50	0.35	0.72	0.91						
KPO	0.39	0.44	0.36	0.19	0.34	0.39	0.77					
OPO	0.27	0.23	0.35	0.32	0.30	0.30	0.17	0.78				
EXM	0.12	0.09	0.03	0.02	0.07	0.21	0.21	0.22	0.79			
IJR	0.14	0.03	0.05	0.07	0.07	0.14	0.11	0.24	0.48	0.78		
IDR	0.47	0.41	0.38	0.30	0.50	0.66	0.37	0.33	0.40	0.38	0.85	
INM	0.49	0.51	0.49	0.33	0.66	0.72	0.42	0.35	0.25	0.26	0.70	0.89

**TABLE 5:** Discriminant validity – Heterotrait-Monotrait (HTMT) ratio for the measurement model.

	PEM	PEC	PESD	PEI	KSB	KSI	KPO	OPO	EXM	IJR	IDR
PEC	0.58										
PESD	0.48	0.67									
PEI	0.24	0.34	0.52								
KSB	0.51	0.49	0.56	0.48							
KSI	0.55	0.60	0.53	0.36	0.76						
KPO	0.44	0.50	0.41	0.22	0.39	0.43					
OPO	0.28	0.24	0.36	0.36	0.32	0.30	0.19				
EXM	0.14	0.13	0.07	0.03	0.09	0.22	0.25	0.27			
IJR	0.14	0.08	0.08	0.13	0.09	0.14	0.13	0.30	0.68		
IDR	0.53	0.46	0.43	0.33	0.56	0.73	0.43	0.34	0.46	0.44	
INM	0.53	0.55	0.53	0.36	0.71	0.78	0.48	0.38	0.28	0.27	0.78

We modeled the user PE construct as a second-order formative construct formed by four first-order reflective constructs. Thus, examinations of the significant weights and multicollinearity were performed (Petter et al., 2007). The results showed that the weights were all significant (*p* < 0.001). To evaluate for multicollinearity, we conducted a variance inflation factor (VIF) test to ensure the validity of the formative construct. The results indicated that the VIFs for the first-order indicators of user PE (ranging from 1.32 to 1.96) were less than the cutoff value of 3.3 (Petter et al., 2007), and the magnitude of the error terms were small. Therefore, high multicollinearity was not a serious concern. The results also confirmed that user PE could be conceptualized as a second-order factor.

To reduce potential common method bias, several procedures were implemented (Podsakoff et al., 2003; Lin and Huang, 2010). First, we arranged our survey items of the dependent and independent variables (i.e., latent constructs of interest) in our research model in a random manner and purposely presented the measures of the dependent variables behind rather than preceding those of the independent variables. Second, the reverse items were designed purposely include in our questionnaire to check whether a respondent, either unintentionally or deliberately, had a systematic answering pattern. Third, the respondents’ responses were anonymous and confidential to encourage comfortable and honest participation in this survey.

Regarding statistical remedies, we performed a Harman’s single-factor test to assess for potential common method variance (CMV) bias. In this approach, all survey items were loaded into the principal component factor analysis using an unrotated factor solution. The results showed the presence of 12 distinct factors with eigenvalues >1.0, rather than a single factor, with the first factor explaining only 29.6% of the variance. Thus, CMV was not considered a serious issue in this research.

### Hypotheses Testing

**FIGURE 2:**
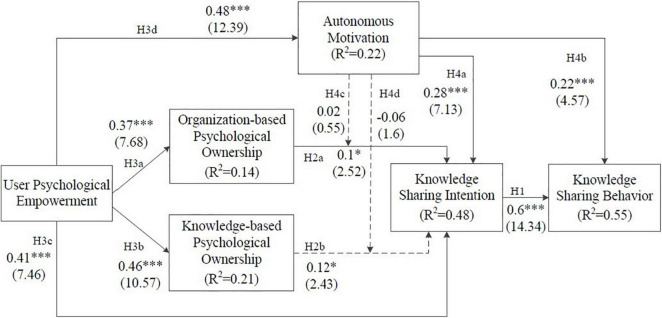
Hypotheses testing results. 1: Standardized path coefficients (β) are reported (*t* values in parentheses). 2: *p < 0.05; ^***^p < 0.001. 3: Supported; not supported - ->.

We used the structural model to examine the significance of the proposed hypotheses. To evaluate the fit of the structural model, the coefficient of determination (R^2^) for endogenous constructs (Wetzels et al., 2009) was first assessed. The results depicted in Figure 2 indicate that a substantial proportion of the variance of the endogenous latent constructs could be explained by the model.

Henseler et al. (2014) presented the standardized root mean square residual (SRMR) as a goodness of fit measure for PLS, which can be utilized to assess the potential issue of model misspecification. Accordingly, we examined the SRMR, which reflects the difference between the empirical model and the model-implied correlation matrix, where a lower SRMR indicates a better fit of the theoretical model (Hair et al., 2017). The results showed that the value of the SRMR (0.04) was less than the threshold value of 0.08 (Hu and Bentler, 1999), indicating that our structural model had a good fit. Finally, we checked the Stone-Geisser’s Q^2^ value using the blindfolding procedure to evaluate the predictive capability of the proposed model (Hair et al., 2017). The results revealed that the Q^2^ values of OPO, KPO, autonomous motivation, KSI, and KSB were equal to 0.14, 0.21, 0.22, 0.47, and 0.54, respectively, all of which were greater than zero, indicating that all endogenous constructs in the proposed model had sufficient predictive relevance (Hair et al., 2017). Overall, the inspection results supported the fit of the structural model.

Consequently, the hypotheses were examined through the bootstrapping procedure with 343 cases and 5,000 resamples. The results are shown in Figure 2.

These results showed that the hypotheses were largely supported by the data, except for H2b, H4c, and H4d. Contrary to our expectations, the statistically significant result for H2b (*p* < 0.05) indicated that KPO has a positive effect on KSI. Meanwhile, it was found that autonomous motivation had neither a significant moderating effect on the relationship between OPO and KSI (*p* > 0.10) nor did it have a moderating effect on KPO and KSI (*p* > 0.10). Thus, H4c and H4d were rejected. The results of our hypotheses testing are presented in Table 6 and Figure 2.

**TABLE 6:** Results of the hypotheses testing.

Hypothesis	Relationship	Path Coefficient	*t*-value	Decision
H1	KSI- > KSB	0.6	14.34	supported
H2a	OPO- > KSI	0.1	2.52	supported
H2b	KPO- > KSI	0.12	2.43	Not supported
H3a	User PE- > OPO	0.37	7.68	supported
H3b	User PE- > KPO	0.46	10.57	supported
H3c	User PE- > KSI	0.41	7.46	supported
H3d	User PE- > ANM	0.48	12.39	supported
H4a	ANM- > KSI	0.28	7.13	supported
H4b	ANM- > KSB	0.22	4.57	supported
H4c	ANM*OPO- > KSI	0.02	0.55	Not supported
H4d	ANM*KPO- > KSI	−0.06	1.6	Not supported

*ANM, autonomous motivation.*

Furthermore, we also examined the mediating effects of the influencing factors on KSI and KSB by adopting the method of Sobel test using the bias-corrected (BC) bootstrapping approach Preacher and Hayes (2004). To be specific, we adopted the macro for the statistical software SPSS developed by Preacher and Hayes (2008) to conduct the mediation analyses. This macro is available on the Internet^1^. In these examinations, the lower and upper bounds of the BC 95% confidence interval (CI) were calculated using 5,000 bootstrapping samples drawn from 343 cases (the same size as the original sample) with replacements.

The results indicated the indirect effect of user PE on KSI via autonomous motivation to be positive (0.13) and that the 95% CI did not contain zero (ranging from 0.09 to 0.18), confirming the significance of this mediating effect. Similarly, user PE can indirectly influence KSI via KPO and OPO. Table 7 shows the results of the examination of mediating effects. Regarding the determination of the types of those mediating effects, because the indirect and direct effects were significant and pointed in the same direction, they could be categorized into complementary mediation (Zhao et al., 2010).

**TABLE 7:** Results of the examination of mediating effects.

Variables			Bias-corrected confidence intervals (CI)			Result
IV	M	DV	Indirect effect	95% Lower CI	95% Upper CI	
User PE	KPO	KSI	0.05	0.01	0.10	Complementary mediation
User PE	OPO	KSI	0.04	0.01	0.07	Complementary mediation
User PE	ANM	KSI	0.13	0.09	0.18	Complementary mediation
User PE	ANM	KSB	0.04	0.01	0.08	Complementary mediation
User PE	KSI	KSB	0.31	0.24	0.38	Complementary mediation

*IV, independent variable; M, mediator variable; DV, dependent variable; ANM, autonomous motivation.*

Regarding the influences of the control variables, the results showed that gender (β = −0.03; *t* = −0.96), age (β = −0.05; *t* = −0.85), education (β = 0.01; *t* = 0.28), and tenure (β = 0.12; *t* = 1.87) did not significantly affect KSB (*p* > 0.05), indicating that our hypotheses testing results remained the same when controlling for the effects of age, gender, education, and tenure.

Overall, these results indicated that user PE, OPO, KPO, and autonomous motivation accounted for 48% of the variance of the KSI construct. All constructs that directly or indirectly influenced KSB accounted for 55% of its variance.

## Discussion and Implications

### Discussion

First, this study confirms the significant positive influence of KSI on actual KSB, and is supported by TRA and TPB, which deem KSI to be the closest determinant of behavior (Ajzen and Fishbein, 1977; Ajzen, 1991). Second, these results show that user PE has significant positive effects on OPO, KPO, KSI, and autonomous motivation, indicating that user PE is an essential ingredient for KS. In the context of HIS usage, employes may perceive the value and impact of HIS, which makes it possible for them to efficiently perform tasks and collaborate with others to solve problems, leading to a proactive orientation toward the use of HIS by trying more of its features. Meanwhile, empowered employes are granted powers and authority to make decisions and thus hold a degree of responsibility, autonomy, and decisiveness (Bendermacher et al., 2019), leading them to exhibit self-determined orientations toward continuous improvement of HIS functions. These orientations can raise employes’ degree of autonomous motivation regarding KSI for the purpose of achieving the collective goals of their healthcare organizations (Gagne, 2009; Wang, 2016). Moreover, empowered employes may have high confidence in their competence (Spreitzer, 1995; Benton and Magnier-Watanabe, 2014; Dust et al., 2014), which strengthens their self-identity and increases their sense of belongingness to the target of ownership (i.e., organization and knowledge). This satisfies the three basic needs of PO (efficacy, self-identity, and belongingness), which cultivate feelings of PO.

Third, we found that OPO has a significant positive effect on KSI, which implies that employes with a higher degree of feelings of OPO may exhibit a stronger sense of responsibility and accountability to their organizations. This strengthens the close relationship between employes and organizations, resulting in more positive work-related outcomes (e.g., exhibiting higher levels of intention to share knowledge). Specifically, KPO was found to significantly affect KSI, which was contrary to the findings of a previous study (Li et al., 2015). A plausible explanation for this finding is the unique nature of the medical industry. Healthcare professionals have essential valuable and technical knowledge, and they may assume ownership of such knowledge, thus perceiving a higher level of KPO. Such feelings of possession reflect the awareness, beliefs, and thoughts of employes (Pierce et al., 2003). Furthermore, the target of ownership (knowledge) can be regarded as a symbolic expression of the self (Pierce et al., 2001, 2003), in turn strengthening self-identity. However, when the value of one’s knowledge is recognized by others, this may enhance and affirm self-worth and feelings of respect and pleasure when interacting with others at work, which in turn promotes willingness to share knowledge.

Finally, we observed the interactions between these influencing factors. This finding unexpectedly showed that autonomous motivation does not amplify the impact of OPO on KSI. This was attributed to the fact that employes who have high feelings of OPO may have a deep affectional investment in the organization, reflecting a sense of belonging, responsibility, and accountability to the organization (Avey et al., 2009; Dawkins et al., 2017; Pittino et al., 2018). This can be stimulated to produce more beneficial outcomes for the organization, where OPO is not leveraged by autonomous motivation. On the other hand, healthcare professionals with higher KPO tendencies may consider themselves to be the owners of knowledge. They may master knowledge, have more control over knowledge, and exercise discretion as to whether to share it, which implies that they have a certain degree of self-determination. Therefore, for those with have high levels of autonomy, autonomous motivation does not have a strong influence on the relationship between KPO and KSI.

In addition, the results show that autonomous motivation is directly positively associated with KSI and KSB, as expected. This implies that fostering autonomous motivation in employes is critical for facilitating KSB because when they sense a high level of autonomy, they are likely to inherently value KSB and thus engage in KSB.

### Implications for Research

This study contributes to the KS and IS literature in several significant ways, as summarized below. First, this study links employes’ psychological motivation, cognition, and behavior by integrating the PE and PO perspectives with SDT, which offers a broader perspective on KSB in healthcare organizations. This offers a comprehensive understanding of how numerous variables (user PE, PO, autonomous motivation, and KSI) affect employes’ KSB, and remedies the lack of theoretical understanding of the interactions among these predictors, which can serve as the foundation for future theorizing initiatives for KSB.

Second, the effect of user PE on KS is another unique aspect of this study and contributes to the application of PE theory to the IS domain. HISs comprise one of the most important ISs in healthcare organizations. They provide support that helps employes efficiently perform tasks and improve the quality and efficiency of healthcare services. This research conceptualized user PE as the extension of a specific type of PE in the context of HIS use. It reflects employes’ motivational orientations toward the use of HISs at work and the authority necessary to exert the full potential of working systems. User PE plays an important role in increasing feelings of PO, including OPO and KPO, and nurtures the KSI of employes. We provided evidence of the significant effect of autonomous motivation on KS, and found that increased levels of user PE can lead to a greater degree of autonomous motivation of healthcare professionals regarding KSB, which has not been empirically examined in the existing literature. These findings shed additional light on active motivational orientations (e.g., user PE and autonomous motivation) that are beneficial to successful KS and conducive to developing new insights into the motivational mechanisms that support the applicability and extension of PE theory and SDT.

Third, this study enriches our understanding of PO (OPO and KPO) regarding KSB in healthcare organizations and contributes to the KS literature by addressing existing gaps in previous studies where the effect of KPO on KSI remains mixed. We provided evidence supporting the positive relationship between PO and KSI. Specifically, we found the positive side of KPO in healthcare organizations, which reflected the unique characteristics of healthcare professionals. There may be differences in this issue across various workplace contexts. This empirical finding supports future theoretical development intended to deepen the understanding of employes’ behavioral patterns regarding KS in healthcare organizations, which has not been adequately addressed in the literature.

Finally, this research goes beyond the traditional dichotomous view of motivation (e.g., intrinsic/extrinsic) by adopting the autonomous motivation construct. The measurement was represented by a compound RAI that encompasses motivation on a controlled-to-autonomous continuum, rather than using proxies such as altruism or financial rewards. This construct can clearly reflect the relative autonomy associated with individual behavioral motivation (Cockrell and Stone, 2010) and makes it possible to better understand the overall effects of autonomy on employe KSB. These results demonstrated that autonomous motivation can facilitate appropriate employe behavior (e.g., KSB) and endorse SDT perspectives that effectively interpret the direct impact of motivation on behavior (Deci and Ryan, 2000) and contributes to investigations of conceptualized autonomous motivation in the medical field, which is a relatively less studied context related to KS.

### Implications for Practice

In the following, we provide important managerial implications related to efforts to enhance employe KSI and lead to effective KS practices in healthcare organizations.

First, in a patient-centered care environment, relying solely on single-professional care may not be sufficient to address all patient issues. KS and integration across different specialties are key issues. However, user PE and autonomous motivation are effective in promoting voluntary behavior. Managers should promote multi-professional knowledge integration to obtain well-balanced inter-professional collaboration through empowerment and autonomy. Multi-specialist teams can be empowered by giving them increased responsibility and autonomy by which to devise healthcare plans for patients with high homogeneity and establish clinical pathways through discussion and high levels of involvement. This may enhance user competence related to user PE and exert and impact from HIS usage that will improve HIS functions so they meet task requirements, in turn generating higher levels of mutual knowledge and experience sharing among employes.

Second, these findings indicated that PO, whether OPO or KPO, encourages employe KSI, making KSB smoother. We suggest building a favorable work environment that leads to satisfaction of efficacy, self-identity, and a sense of belongingness to cultivate the sense of PO. For example, employes can take advantage of on-the-job education and training, as well as seminars, thus increasing opportunities for learning and absorbing new knowledge and improving professional competence and confidence in their ability to respond to the rapidly changing healthcare environment. Moreover, publicly announcing a list of honors for KS contributors who are recognized and praised may lead to their being respected and highly accomplished, in turn strengthening their sense of self-identity. Also, more opportunities for participation and communication can be offered to encourage interaction among employes through various activities, such as health promotion, patient safety awareness, and healthcare quality improvement campaigns. This may build consensus and promote feelings of responsibility for the targets of PO, result in an increased sense of belonging. Additionally, by enhancing an intimate mentor-protege relationship through close interaction, the transformation that takes place due to experience, knowledge, and emotional care will help form a positive teaching and learning environment, in turn promoting KS practices.

Finally, self-efficacy and enjoyment will have a significant effect on the use of electronic knowledge repositories (Jiang and Xu, 2020), and an increase in the enjoyment of work could encourage intrinsic motivation (Thomas and Velthouse, 1990; Gagne, 2009). This may lead to high levels of user PE and autonomous motivation, allowing employes to use HISs seamlessly into their daily work, which in turn will increase their willingness to share knowledge. However, not all work is interesting, so managers can make efforts to enhance accessibility to workplace communication by providing more effective information technology tools and by increasing their novelty and increasing employe enjoyment, while taking into account that employes need sufficient choices and autonomy in the workplace. For example, video conferencing systems that can instantly share various information such as X-rays, pathology files, videos, and texts, can be employed in bidirectional referral communications, telemedicine, distance learning, and lectures, to practice real-time KS in different locations. Being free from space constraints will promote more autonomous discussions and interactions. Also, the provision of e-learning platforms gives healthcare professionals the freedom to decide when to acquire work-related information automatically. On the other hand, virtual reality (VR) is an emerging technology developed with computer multimedia technology in recent years. The VR environment provides users with an immersive experience, which creates more opportunities for learning and KS. This not only intensifies user competence and user PE, but also familiarizes healthcare professionals with actual treatment scenarios that will reduce mistakes. Further, encouraging the use of artificial intelligence (AI) that assists in predicting adverse events, alleviates anxiety related to clinical care through the active participation of healthcare professionals, and continuously contributes knowledge that leads to high accuracy. Doing so may drive more efficient knowledge dissemination and absorption in an extremely interesting way, making employes feel comfortable and improving their skill level and competence by which to control their surroundings, increasing inspiration and upward mobility. This may motivate positive behavior in organizations such as KSB.

## Conclusion and Future Research

This study provides empirical evidence by integrating the PE and PO approaches with the SDT, with a focus on KS in healthcare organizations, which has not been heavily studied. We explored the intensity and significance of numerous factors, including user PE (a special type of PE in the context of HIS usage), OPO, KPO, autonomous motivation, and the effects of KSI on KSB, to better understand the entire employe KSB picture, in order to find new ways to boost, support, and improve KSB in the workplace. These findings revealed a positive and significant impact of user PE, PO (including OPO, KPO), and autonomous motivation on KSI and KSB, offering valuable insights that will help managers facilitate effective, efficient KS practices. Although these results contribute to theory and practice, there are still some limitations that suggest possible directions for further research.

First, the survey data in this study were collected from employes of healthcare organizations in Taiwan, which may have a limitation related to the regional culture, thus affecting the generalization of the results. Depending on the cultural context, feelings of PO may vary (Dawkins et al., 2017). Thus, it is recommended that future research explore cross-cultural differences in PO.

Second, this study categorized PO into OPO and KPO, which are fruitful contributions to advancing an understanding of PO and its effects, but may have limited the scope of the investigation of PO. Further studies are suggested to examine these targets of PO toward different foci beyond employe knowledge and organizations, such as projects, specific roles, and workspaces, so as to enrich the empirical research in the PO literature.

Third, these findings reveal that user PE has significantly positive effects on autonomous motivation, PO, and KSI, thus playing an important role in KSB. PE theory was applied to the context of HIS usage in healthcare organizations, where user PE was viewed as a specific type PE in order to deepen the understanding of KS practices among healthcare professionals. However, different industries may implement different IS to improve performance. Future research could apply PE to different scenarios due to different industry characteristics.

Finally, this study investigated the factors influencing KSB in healthcare organizations, and the results indicated positive effects on KSB. It is unclear whether the factors that boost KS may also decrease the likelihood of employes withholding knowledge. For future researchers, this may be a good topic to advance the understanding of the opposite sides of KSB.

## Data Availability Statement

The raw data supporting the conclusions of this article will be made available by the authors, without undue reservation.

## Ethics Statement

Ethical review and approval was not required for the study on human participants in accordance with the local legislation and institutional requirements. The patients/participants provided their written informed consent to participate in this study.

## Author Contributions

S-YW contributed to develop the theoretical framework, data collection, data analysis, and manuscript writing. W-TW contributed to design the theoretical framework, review and edit the manuscript, and overall design. M-HH contributed to conduct data collection and data analysis processes. All authors contributed to the article and approved the submitted version.

## Conflict of Interest

The authors declare that the research was conducted in the absence of any commercial or financial relationships that could be construed as a potential conflict of interest.

## Publisher’s Note

All claims expressed in this article are solely those of the authors and do not necessarily represent those of their affiliated organizations, or those of the publisher, the editors and the reviewers. Any product that may be evaluated in this article, or claim that may be made by its manufacturer, is not guaranteed or endorsed by the publisher.

## Footnotes

1
http://afhayes.com/spss-sas-and-r-macros-and-code.html

## Abbreviations

HISs: health information systems
KM: knowledge management
KS: knowledge sharing
KSB: knowledge sharing behavior
KSI: knowledge sharing intention
PO: psychological ownership
KPO: knowledge-based psychological ownership
OPO: organization-based psychological ownership
PE: psychological empowerment
PLS: Partial Least Square
RAI: relative autonomy index
SDT: self-determination theory
SEM: structural equation modelings.
