# Supplementary material for: Knowledge Sharing Among Healthcare Practitioners: Identifying the Psychological and Motivational Facilitating Factors
Source: Front Psychol. 2021 Dec 14;12:736277. doi: 10.3389/fpsyg.2021.736277 (PMC8712574; doi:10.3389/fpsyg.2021.736277)
Supplement: Supplementary file 1 [file Data_Sheet_1.pdf]

Appendix. Questionnaire items.

Constructs	Dimensions	Items	Questions
User Psychological empowerment in the context of HIS usage *(Spreitzer (1995)*	Meaning of HIS usage	PEM1	The work I do is very important to me.
		PEM2	My job activities are personally meaningful to me.
		PEM3	The work I do is meaningful to me.
	User competence	PEC1	I am confident about my ability to do my job.
		PEC2	I am self-assured about my capabilities to perform my work activities.
		PEC3	I have mastered the skills necessary for my job.
	User self-determination	PESD1	I have significant self-determination in determining how I do my job.
		PESD2	I can decide on my own how to go about doing my work.
		PESD3	I have considerable opportunity for independence and freedom in how I do my job.
	Impact of HIS usage	PEI1	My impact on what happens at work is large.
		PEI2	I have a great deal of control over what happens at work.
		PEI3	I have significant influence over what happens at work.
Autonomous motivation *(Cockrell and Stone (2010)*			I share my professional knowledge with others
	External motivation	EXM1	Because I will get in trouble if I do not. (discarded)
		EXM2	Because it is what I am supposed to do.
		EXM3	Because I know that I will get a reward for doing so.
		EXM4	Because it would harm my relationships with others if I did not share my professional knowledge with others.
	Introjected regulation	IJR1	Because I want my supervisor to think I am a good employee.
		IJR2	Because I feel bad about myself if I do not.
		IJR3	Because I want people to like me.
		IJR4	Because it bothers me when I do not.
	Identified regulation	IDR1	Because I want others to understand what I know.
		IDR2	Because it is important to me to share knowledge.
		IDR3	Because I think it is important to help others at work.
		IDR4	Because I believe it is an important personal attribute to share what I know

			with others.
	Intrinsic motivation	INM1	Because it is fun.
		INM2	Because I enjoy doing so.
		INM3	Because of the happiness I feel when I share knowledge with others.
		INM4	Because it is interesting and satisfying to share my professional knowledge.
Organization-based Psychological ownership (OPO) *(Van Dyne and Pierce (2004)*		OPO1	This is MY organization.
		OPO2	I sense that this organization is OUR hospital.
		OPO3	I feel a very high degree of personal ownership for this organization.
		OPO4	I sense that this is MY hospital.
		OPO5	This is OUR hospital.
		OPO6	Most of the employees that work for this organization feel as though we own the hospital.
		OPO7	It is hard for me to think about this organization as MINE. (discarded)
Knowledge-based Psychological ownership (KPO) *(Van Dyne and Pierce (2004)*		KPO1	This is MY knowledge.
		KPO2	I sense that this knowledge is OUR.
		KPO3	I feel a very high degree of personal ownership for this knowledge.
		KPO4	I sense that this is MY knowledge.
		KPO5	Most of the employees feel that the knowledge used in the work as though we own.
		KPO6	It is hard for me to think about this knowledge as MINE. (discarded)
Knowledge sharing intention (KSI) *(Bock et al. (2005)*		KSI1	I will share my work reports and official documents with members of my organization more frequently in the future.
		KSI2	I will always provide my manuals, methodologies and models for members of my organization.
		KSI3	I intend to share my experience or know-how from work with other organizational members more frequently in the future.
		KSI4	I will always provide my know-where or know-whom at the request of other organizational members.
		KSI5	I will try to share my expertise from my education or training with other organizational members in a more effective way.
Knowledge		KSB1	I frequently participate in the knowledge

sharing behavior (KSB)<br><br>*(Hsu et al. (2007)*		sharing activities in the hospital.
	KSB2	I usually spend a lot of time conducting knowledge sharing activities in the hospital.
	KSB3	I usually actively share my knowledge with my colleagues.
	KSB4	When discussing a complicated issue with my colleagues, I am usually involved in the subsequent interactions.
	KSB5	I usually involve myself in discussion of various topics rather than specific ones in the hospital.
